# Acoustic Streaming Efficiency in a Microfluidic Biosensor with an Integrated CMUT

**DOI:** 10.3390/mi14051012

**Published:** 2023-05-08

**Authors:** Donatas Pelenis, Gailius Vanagas, Dovydas Barauskas, Mindaugas Dzikaras, Marius Mikolajūnas, Darius Viržonis

**Affiliations:** Panevėžys Faculty of Technologies and Business, Kaunas University of Technology, 37164 Panevėžys, Lithuania

**Keywords:** microchannel, acoustic streaming, CMUT, micro particles, micro-PIV

## Abstract

The effect of microchannel height on acoustic streaming velocity and capacitive micromachined ultrasound transducer (CMUT) cell damping was investigated. Microchannels with heights ranging from 0.15 to 1.75 mm were used in experiments, and computational microchannel models with heights varying from 10 to 1800 micrometers were simulated. Both simulated and measured data show local minima and maxima of acoustic streaming efficiency associated with the wavelength of the `bulk acoustic wave excited at 5 MHz frequency. Local minima occur at microchannel heights that are multiples of half the wavelength (150 μm), which are caused by destructive interference between excited and reflected acoustic waves. Therefore, microchannel heights that are not multiples of 150 μm are more favorable for higher acoustic streaming effectiveness since destructive interference decreases the acoustic streaming effectiveness by more than 4 times. On average, the experimental data show slightly higher velocities for smaller microchannels than the simulated data, but the overall observation of higher streaming velocities in larger microchannels is not altered. In additional simulation, at small microchannel heights (10–350 μm), local minima at microchannel heights that are multiples of 150 μm were observed, indicating the interference between excited and reflected waves and causing acoustic damping of comparatively compliant CMUT membranes. Increasing the microchannel height to over 100 μm tends to eliminate the acoustic damping effect as the local minima of the CMUT membrane swing amplitude approach the maximum value of 42 nm, which is the calculated amplitude of the freely swinging membrane under the described conditions. At optimum conditions, an acoustic streaming velocity of over 2 mm/s in a 1.8 mm-high microchannel was achieved.

## 1. Introduction

One of the most pressing issues in public health is the need for fast and accurate diagnostic tests for infectious diseases. Acoustic microfluidic systems can be used to manipulate and analyze small volumes of biological samples, such as blood, urine, or saliva, with high precision and sensitivity [1,2,3,4,5]. The use of acoustic streaming in microchannels can enhance the performance of these systems by enabling separation of the sample components, mixing of them in fluids, and transporting extremely small analyte quantities. These are critical steps in many diagnostic assays.

Recently, we have developed a capacitive micromachined ultrasound transducer (CMUT) integrated real-time microfluidic biosensor [5], which is also capable of controlling the biochemical interaction rate via acoustic streaming [4]. Microchannels with integrated CMUTs employ two types of ultrasonic waves that can induce acoustic transport. These are bulk acoustic waves (BAW) and surface acoustic waves (SAW), which can express themselves as Rayleigh or Scholte waves [6]. The choice of the acoustic transport mechanism is directly related to the optimization of microchannel materials, configuration, and design. SAWs are generally more effective in microchannels, which have a small height-to-width ratio, while BAWs, which leak from the surface to the bulk of the liquid, can be more effective for acoustic streaming in microchannels with heights larger than the half wavelength [7,8,9,10]. However, SAW-based acoustic streaming typically requires a SAW transducer outside the microchannel, which drastically increases the device footprint. The same issue is found in other microfluidic devices with active acoustic or photoacoustic streaming produced by lasers or piezoelectric transducers [11,12].

CMUTs were first used in microfluidic applications to manipulate fluids and sense their physical properties using Scholte waves, which propagate along the membrane-fluid interface instead of standard pressure waves that travel into the fluid bulk [9]. Recently, CMUT-induced Scholte waves have been utilized to control biochemical adsorption and interaction rates [4]. Although a CMUT acoustic source has been used to manipulate a microparticle into the center of a CMUT cell, it was not optimized for array operation or concurrent manipulation of multiple particles [13]. A simulation of a CMUT-integrated standing wave resonator was described, but it was not fully fabricated or experimentally verified, possibly due to stringent dimensional tolerances and material constraints in microchannels that act as acoustic resonators.

As indicated by previous research [9,14], CMUT-operated acoustic streaming has significant differences from similar cases driven by piezoelectric devices. This is primarily because CMUTs can be easily designed for two-phase, directional control of the acoustic transport. Furthermore, the structure and mode of operation of CMUT cells make them more susceptible to complex dynamic interactions in the confined acoustic space of microchannels. Therefore, this work explores the efficiency of acoustic streaming, produced by BAW-generating CMUTs, within a wide range of microchannel heights, from ¼ to 5 wavelengths of the acoustic waves.

## 2. Materials and Methods

### 2.1. Basis for Computational Modeling

Acoustic streaming is a second-order (non-linear) acoustic effect that can be described by the introduction of non-linear terms in the Navier–Stokes equations. For a non-viscous fluid with no deviatoric stress (no difference between the stress tensor and the hydrostatic pressure tensor), the steady-state, time-averaged streaming flow in an acoustic field will be described by the following equations [15]:

(1) $$0=-\nabla \cdot \left(\rho_{0}v_{2}\right),$$

(2) $$0=-\nabla \cdot \sigma_{2}+f_{ac},$$

with the mean fluid velocity of $v_{2}$, as the fluid density, $\sigma_{2}$ as the stress tensor, and $f_{ac}$ the acoustic force, which, in this case, depends on the gradient of the acoustic pressure field ∇ represents the gradient operator.

The $f_{ac}$ can be expressed as:

(3) $$f_{ac}=\frac{-1}{4}{\left|p_{1}\right|}^{2}\nabla \kappa_{s,0}-\frac{1}{4}\left|v_{1}\right|\nabla \rho_{0},$$

where $p_{1},v_{1}$ are the ultrasonic pressure and velocity, respectively, and $\kappa_{s,0}$ is the isentropic compressibility of the fluid.

### 2.2. Continuous Acoustic Flow Model

**Figure 1:**
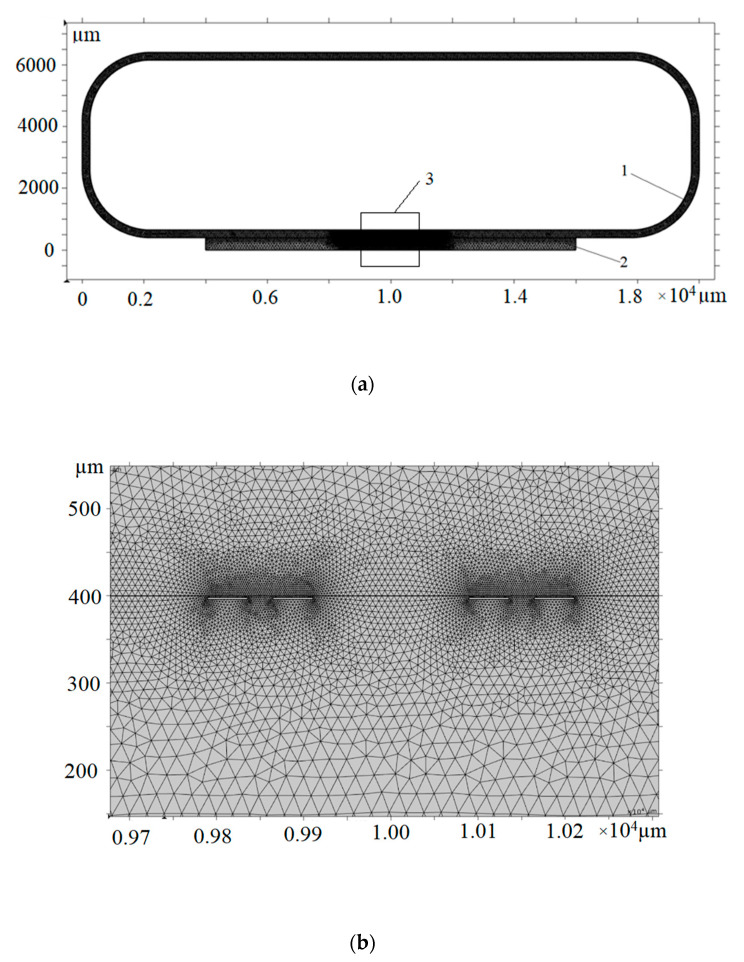

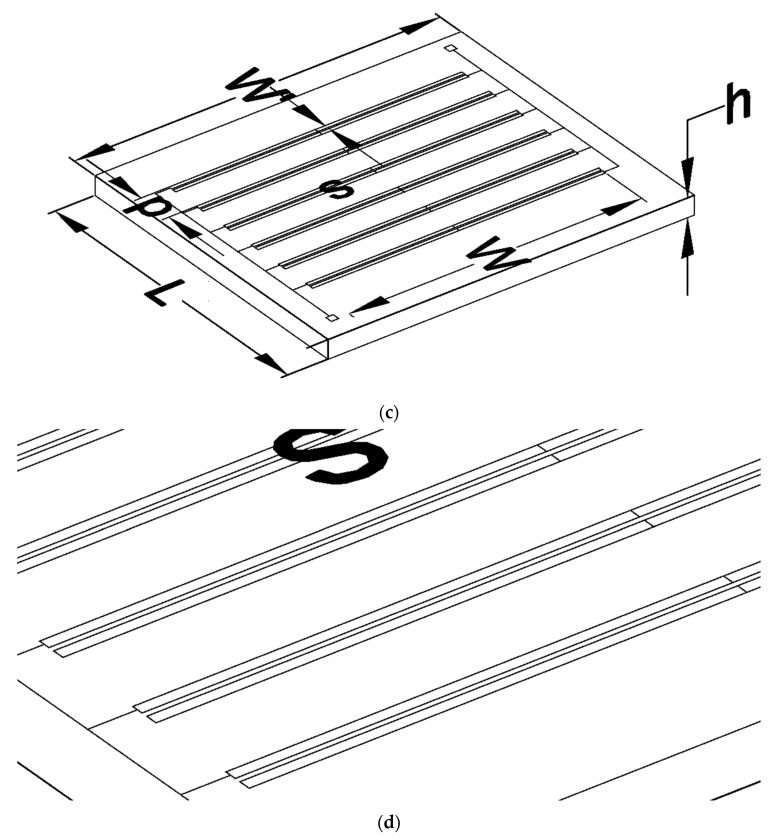
Illustration of the geometry of the continuous acoustic flow model: (**a**) general geometry of the finite element mesh and its density for the 2D model simulation, where 1 indicates the microchannel in polydimethylsiloxane and 2 is the silicon die with embedded CMUT structure. The structure here is depicted as a sideways profile cut; (**b**) the 2D mesh of a part of the CMUT structure, generated for finite element model simulation use; (**c**) an example schematic illustration of the interdigital CMUT structure in three dimensions (not to scale); (**d**) a zoomed-in version of panel (**c**) for better clarity of the CMUT-based finger structure.

The general geometry of the two-dimensional finite element model used in the simulation is explained in Figure 1. The two-dimensional model simulates the cross-sectional microfluidic flow of a tentative real three-dimensional microfluidic structure and the loop structure in the model is included in the same plane as the rest of the device due to modeling restrictions. In an actual device, the loop structure, if needed, can be constructed in a plane with a horizontal surface with the rest of the microfluidic device, since reproducing the loop structure as designed in this simulation can be challenging and unnecessary. The purpose of the loop structure is to ensure a closed microchannel and satisfy the required simulation conditions. The closed microchannel having a perimeter of 50 mm and a width of 3 mm is indicated as position 1 in Figure 1a. The height of the channel varied from 10 to 1800 μm during the simulation. The channel height was calculated from the silicon die surface (Figure 1a, pointer 2) and was extruded upwards from that surface and inwards for the rest of the loop structure.

At the bottom part of the geometry, an interdigital CMUT structure with a double finger structure was embedded into the silicon background plate. In Figure 1b, the part of the mesh illustrating the model area with a cross-section of two double finger structures is shown. CMUTs were simulated as 2 μm thick and 42 μm wide monocrystal silicon membranes excited by a sinusoidal force of 450 μN amplitude and 5 MHz frequency. The idea behind the actual CMUT model geometry is illustrated in Figure 1c. The length of the silicon die L was 12 mm, the thickness was h = 0.5 mm, and the design accommodated 10 pairs of interdigital fingers separated by p = 300 μm pitch, equal to the wavelength of a 5 MHz wave propagating at 1500 m/s. The separation between the centers of the sub-fingers of the double finger structure was s = 75 μm, equal to a quarter of a wavelength. This separation was targeted for a ±90° phase difference between the individual fingers in a pair to maintain the directionality of acoustic pressure. At the same time, dimensions going into the “depth” of the geometry shown in Figure 1a, such as the width of the silicon die W’ and aperture width W of the CMUT array, remained undefined.

The acoustic domain force was computed and applied to the laminar flow interface using the acoustic streaming domain coupling multiphysics feature that couples the pressure acoustics and the frequency domain interface with the laminar flow interface. Comsol Multiphysics 6.1 software was used with corresponding physics add-ons.

### 2.3. Microchannel-Integrated CMUT Design and Fabrication

**Figure 2:**
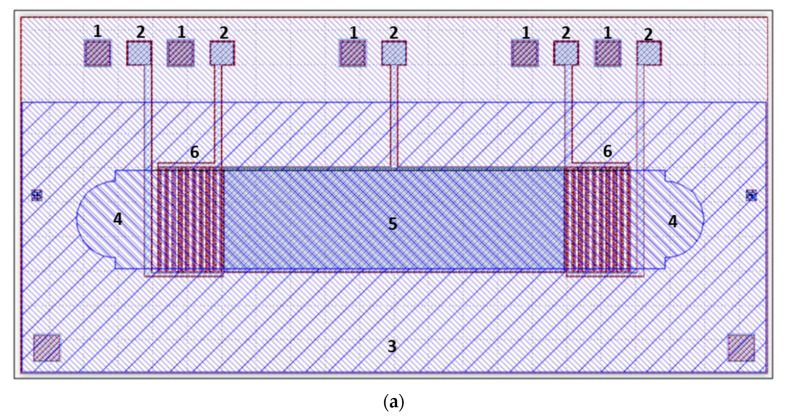

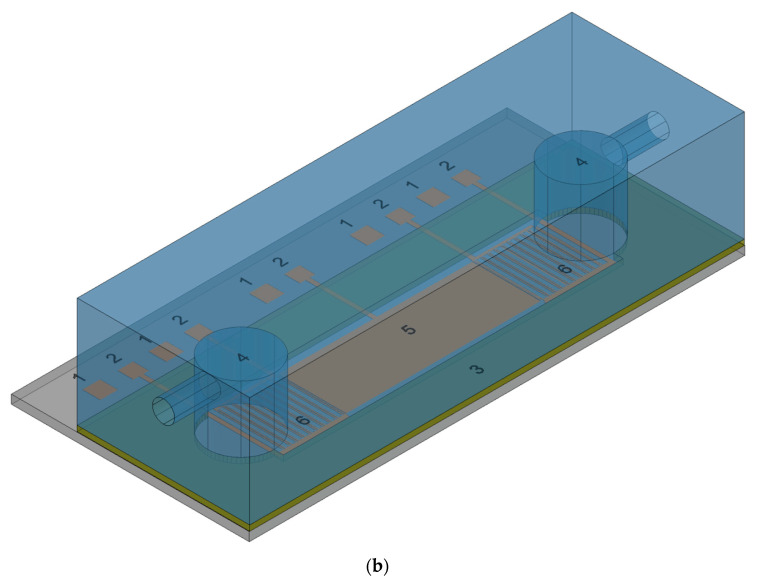
(**a**) Overall view of the CMUT-integrated microchannel design (overall dimensions: 10 by 5 mm): 1—common ground contact pads; 2—upper electrode contact pads; 3—microchannel attachment area; 4—fluid injection and extraction areas; 5—analytic area; 6—interdigital CMUT area; (**b**) a three-dimensional representation of the CMUT-integrated microchannel device layering: gray and brown colors represent the silicon die and the CMUT structures; dark yellow is the attachment area for the PDMS microchannel block; blue is the PDMS microchannel block. The number label meaning is the same as in panel (**a**) of this figure.

The design of the device is illustrated in Figure 2. It is intended to operate in a pitch-catch mode, where a pulse of surface waves is transmitted along the analytical area and the delay time is measured. The CMUT and analytical area are fabricated and assembled using a 0.5 mm thick, 10 by 5 mm monocrystal silicon die as a background. The microchannel is fabricated separately through die-casting of polydimethylsiloxane (PDMS) and then assembled with the CMUT die. The central part of device area 5 is an intended analytical area for surface waves, dedicated to solution analyte interrogation. Two interdigital CMUT arrays (component 6) are fabricated on the ends of the analytical area. The CMUTs were specifically designed for 5 MHz when used in water immersion, with each cell measuring 42 by 3000 μm and having a membrane with a thickness of 2 μm, separated by a 150 nm gap from the background. Microfluidic injection and extraction areas 4 are arranged behind CMUT arrays. The sparsely hatched area around 3 shows the PDMS microchannel attachment zone. Contact pads 1 and 2, which connect the top and bottom CMUT electrodes, are located at the edge of the device and are dedicated to wire bonding.

**Figure 3:**
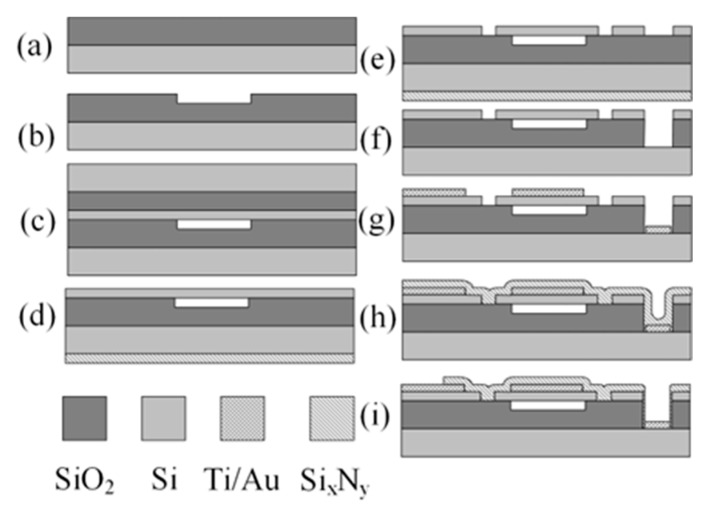
Fabrication of the CMUT structure with the wafer bonding process, cross-sectional view of a single cell: (**a**) thermal oxidation; (**b**) formation of cavities; (**c**) wafer bonding; (**d**) deposition of a thin SixNy layer for backside protection and handle wafer removal using CMP; (**e**) trench etching with DRIE for separating devices and opening contact pads; (**f**) etching of ground openings for bottom electrode and SixNy removal; (**g**) deposition of top and bottom (ground) electrodes; (**h**) thin Si_x_N_y_ PECVD protective layer deposition; (**i**) opening of top and bottom electrode contact pads for wire connections.

The CMUTs were produced using wafer fusion bonding involving various thin layer deposition techniques such as physical and chemical vapor deposition (PCD, CVD) and plasma enhanced vapor deposition (PECVD), along with wet and dry etching to create the device structures and electrodes (Figure 3). The fabrication process involved the oxidation of silicon wafers with thermal oxide (Figure 3a). This was followed by UV photolithography to create cavities within the oxide using buffered oxide etch with the depth being controlled by adjusting the etch duration (Figure 3b). After a standard cleaning procedure, the background wafers were bonded with silicon on insulator (SOI) wafers, forming vacuum gaps in the cavities during the bonding process (Figure 3c). The bonded wafers were then annealed and oxidized in a furnace. An additional thin PECVD silicon nitride layer was added on the backside of the wafer before the SOI handle wafer was thinned down and removed via chemical mechanical polishing (CMP) and chemical etching (Figure 3d). Next, another lithography was done, and the device layer was etched using a deep reactive ion etching process to create trenches between the devices on the wafer and to open areas for contact pads (Figure 3e). Another lithography was done to create the openings in the oxide for ground contact pads, and at the same time, the protective Si_x_N_y_ layer on the backside was etched away (Figure 3f). Furthermore, photolithography was used to create the top and bottom electrodes using a thin layer of Ti/Au, with the underlying titanium layer aiding in better adhesion between the silicon and gold electrodes (Figure 3g). Lastly, a thin layer of PECVD silicon nitride was added as isolation from liquids (Figure 3h), and final lithography was done to open the top and bottom electrode contact pads for wire connections (Figure 3i).

### 2.4. Acoustic Streaming Experiments

**Figure 4:**
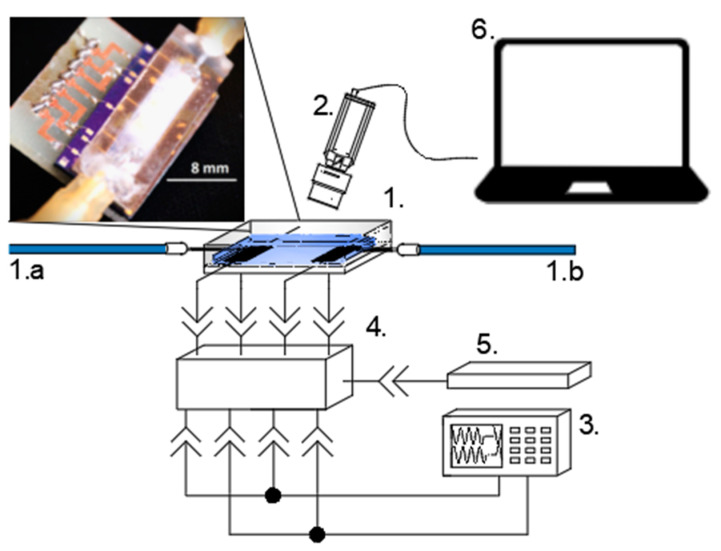
Experimental setup for the micro-PIV measurements. Main components are: 1. Microchannel-integrated CMUT device; 1.a and 1.b. inlet and outlet needles; 2. microscope camera; 3. arbitrary form generator; 4. bias-T; 5. DC bias source; 6. laptop computer. The inset in the top left contains a photo of the assembled device (1.).

The microparticle image velocimetry (micro-PIV) method was used in this research. The experimental setup is illustrated in Figure 4. The microchannel-integrated CMUT (labeled 1 in Figure 4) was supplied with microparticle-containing aqueous solution through medical needles (labeled 1a and 1b in Figure 4) that were attached to the fluid injection and extraction areas (labeled 4 in Figure 3). The 10 µm-diameter polystyrene microparticles (Sigma Aldrich), suspended in deionized (DI) water, were tracked by taking shots with a microscope camera (labeled 2 in Figure 4). The CMUTs were driven by a two-channel arbitrary form generator (labeled 3 in Figure 4). Both interdigital groups on different sides of the analytic area were connected in parallel. Bias-T (labeled 4 in Figure 4) was used to connect the DC bias (5 in Figure 4). Images were captured and processed by a laptop computer (6 in Figure 4).

In the acoustofluidic transport experiments, CMUTs were driven using a two-phase sinusoidal excitation of 5 MHz and 10 V amplitude with a 40 V bias. To control the direction and speed of acoustic streaming, the phase angle was varied between 0 and 2π radians (0°–360°).

For the acoustic streaming velocity calculations, microparticle motion was recorded in video format, and then a series of still images from the captured video data were extracted. The distance between the microparticle positions in still images was measured, and then the distance was divided by the time elapsed between the pair of still images. The speed values were averaged for each selected phase value.

## 3. Results

### 3.1. Model Verification

**Figure 5:**
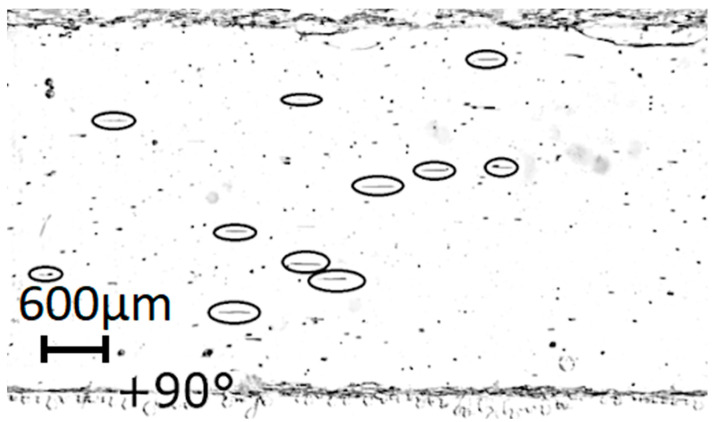
Long exposure micrograph of the 0.2 mm-high microchannel containing polystyrene microparticles. The path of the particles is circled with ellipses. The phase was set to +90° and did not change during the exposure.

The finite element model described in Section 2.1 was verified by taking micro-PIV measurements and comparing the experimental results with the corresponding output of the model. Micro-PIV measurement is illustrated in Figure 5. This micro-PIV image was generated using a 5 s photo capture through the transparent top wall of the microchannel within the region of the analytic area (label 5 in Figure 2). To avoid any residual external flow in the system, the microparticles were introduced just before the CMUTs were excited by a continuous 5 MHz sinusoidal signal of 10 V with a 90° phase difference between the sub-fingers. There were many microparticles within the observed area that remained stationary during the experiment since they adhered to the microchannel walls and the acoustic stream was not powerful enough to move them from their point of attachment. However, suspended particles moved, and they can be identified in the picture as horizontal dashes (circled with ellipses).

The average length of the path is 450 µm, which is equivalent to the 90 µm/s effective velocity of the acoustic stream since the image acquisition took 5 s.

**Figure 6:**
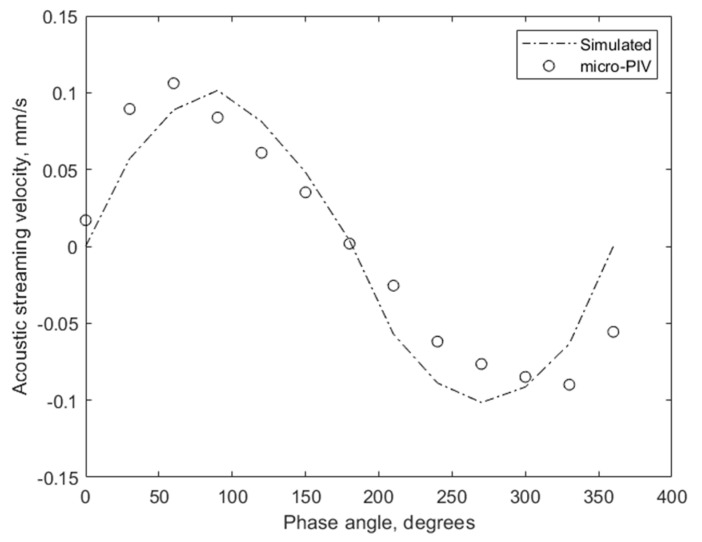
Micro-PIV readings in a 0.2 mm-high microchannel compared with corresponding model output at different phase angles.

During the simulation, the CMUT cells were excited using a 5 MHz sinusoidal force with the amplitude adjusted to produce a surface pressure of 92 kPa. This is the surface pressure of CMUTs excited by a peak-to-peak voltage of 10 V at 5 MHz in water immersion, as measured by the hydrophone ONDA HNP-0200. Similar to the micro-PIV experiment, we varied the velocity and direction of the acoustic streaming during the simulation by changing the phase of excitation of the double fingers from 0° to 360°. We present the results of both the micro-PIV experiment and the computational simulation for comparison in Figure 6.

As can be noted from the comparison of the results, the maximum velocity of the acoustic streaming was accurately simulated, with a measured velocity of 0.1061 mm/s and a simulated velocity of 0.1015 mm/s. However, the maximum values were reached at different phase angles, with 60° for measured data and 90° for the simulated model. This difference can be explained by uncontrolled shifts in the properties of the materials used in the experiment. For instance, the presence of microparticles could have altered the speed of sound and the mechanism of acoustic wave propagation in DI water, while the computational simulation used the properties of uniform and pure water. Other effects that should be taken into account are the influence of friction forces around the walls, microparticles, and temperature dependence. Furthermore, only the average effect of many possible acoustic phenomena, such as the streaming itself, radiation force on particles, wave reflections, and standing waves, is measured. In simulation, we observe only the bulk acoustic waves, which create the pressure difference in the liquid, and the acoustic streaming, but no radiation forces acting over the particles were simulated. Nevertheless, it can be concluded that the computational model’s adequacy is acceptable and that the required reliability of further results can be expected.

### 3.2. Acoustic Streaming Velocity in the Microchannels of Different Height

**Figure 7:**
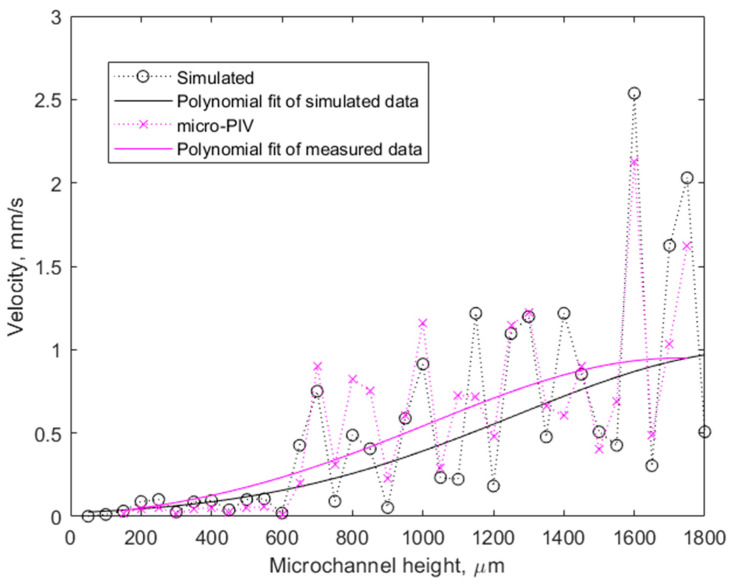
Acoustic streaming velocity in different-height microchannels. Dotted lines have been added to guide the eye between the marked data points.

The dependence of acoustic streaming velocity on microchannel height was investigated using the simulated computational model and micro-PIV experiments. Microchannels of heights varying from 0.15 to 1.75 mm and a constant 3 mm width, which were printed using 3D printing technology, were used for the experiments. In the computational model, the microchannel height was varied from 50 to 1800 micrometers with a 50 µm step. The CMUT excitation parameters were the same as in the previous experiment and simulation, but the phase difference of the sub-fingers excitation was held constant at 90°. Results are illustrated in Figure 7. Dotted lines were added for guidance only and do not illustrate any data between the markers. 

There are many discrepancies between the model and the measurement conditions, such as standing waves and reflections from upper, bottom, and side walls, to account for large deviations between measurement and simulation peak data; however, the general trend is the same in both cases. Both simulated and measured data look quite scattered; however, local minima and maxima can be almost unambiguously associated with the wavelength of the bulk acoustic wave, which is 300 µm for 5 MHz in water. Local minima occur at microchannel heights of 150, 300, 450, 600 µm, etc.—all of them being multiples of the half wavelength. The observed data are the footprint of the complex interferences between the acoustic waves excited by the CMUT structures and their reflections from the microchannel upper wall. In this case, microchannel heights, which are not multiples of 150 µm, are more favorable for more effective acoustic streaming. The destructive interference can decrease the acoustic streaming effect down to 25% or less compared to an adjacent data point, representing only a slightly taller or shorter microchannel. However, the general trend of increasing streaming velocity with taller microchannels is supported by third-order polynomial fits of both simulated and experimental data. This is in agreement with the Hagen–Poiseuille equation used for calculating the pressure drop of a laminar flow through a long cylindrical pipe of constant dimensions. When the volumetric flow rate in this equation is expressed in terms of cross-sectional pipe area and flow velocity, the flow velocity can be calculated as follows:

(4) $$v=\frac{A\Delta p}{8\pi \mu L},$$

where *v* is the flow velocity, *A* is the cross-sectional area of the pipe, Δ*p* is the pressure difference, *μ* is the dynamic viscosity, and *L* is the length of the pipe. In cases where Δ*p*, *μ,* and *L* are constant, as is the case with our simulation and experimental setup, *v* ∝ *A,* and thus with an increase in microchannel height and therefore an increase in the cross-sectional area *A*, the flow velocity *v* increase is expected. The limiting factors for the growth of *v* are only the power output of CMUT devices and the efficiency of the energy transfer into the liquid.

On average, the experimental data show slightly higher velocities for smaller microchannels than the simulated data. However, this difference is insignificant and does not alter the overall observation of higher streaming velocities in larger microchannels.

### 3.3. Acoustic Damping of CMUT Membranes

**Figure 8:**
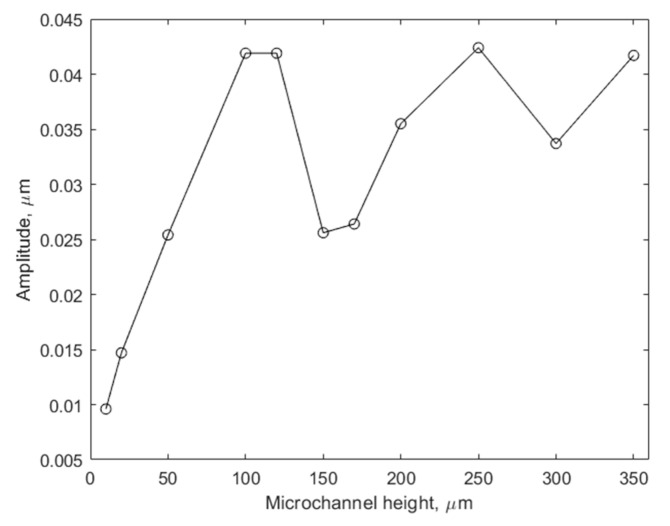
Amplitude of CMUT membrane swing with respect to the microchannel height. The lines between the data points are for guidance only.

Specific attention was paid to the range of small microchannel heights (10–350 µm) to obtain additional support for our hypothesis that microchannel heights at half the wavelength create conditions for destructive interference between the excited and reflected acoustic waves. In this computational simulation experiment, we measured the maximum displacement amplitude of the CMUT membranes relative to the microchannel height. Results are illustrated in Figure 8.

Similar to the results of the acoustic streaming experiment illustrated in Figure 7, local minima can be observed at microchannel heights that are multiples of 150 µm. This can be explained by the interference between excited and reflected waves, which causes acoustic damping of comparatively compliant CMUT membranes. The device is in near-field conditions at the lower end of microchannel heights, where acoustic damping is more prominent. Increasing the height to over 100 μm tends to eliminate the acoustic damping effect as the local swing minima approach the maximum value of 42 nm, which is the calculated amplitude of the freely swinging membrane under the described conditions.

## 4. Discussion

The results demonstrate that microchannels of relatively low height (50–100 µm) commonly used in microfluidic systems are too low for effective acoustic streaming with directly integrated CMUTs. Under these conditions, destructive interference occurs, which can result in the suppression of CMUT membrane movement in smaller microchannels. Additionally, due to destructive interference, unfavorable conditions for acoustic streaming are formed in microchannels whose height is a multiple of half of the acoustic bulk wave wavelength (150 µm in our case), causing a more than 4 times decrease in the speed of acoustic transport. The effect of destructive interference persists even under conditions of larger microchannel dimensions (tested up to 1800 µm), so it is recommended to avoid microchannel dimensions that are multiples of half of the acoustic wavelength. This is corroborated by Devendran et al.’s research on SAWS generated outside the microchannel [16]. Their SAW energy is transmitted into the bulk much more indirectly, but they still observe a strong correlation between channel height as a multiple of the selected wavelength fraction and the induced acoustic streaming velocity. However, they do not extend their study into channel heights as multiples of whole wavelengths, and their setup does not contain the transducers inside the microchannel, making the device less compact when compared to the setup demonstrated in this paper. Furthermore, our design relies on direct BAW generation, possibly allowing for more efficient device construction.

Similarly, dependence on microchannel clearance, wavelength, and streaming efficiency was observed by Lin et al. in their side profile microstructure analysis [17]. Their simulation of the gap between the top of a vibrating microstructure and the microchannel’s upper wall elucidates the optimal microstructure height and gap for streaming velocity and streaming area in the context of microstructures. Relatedly, they too observe that streaming efficiency increases with selected frequencies when the gap matches multiples of the wavelength, further demonstrating the importance of correct channel height in microfluidics design.

As a general trend, taller microchannels are more favorable for higher acoustic streaming velocities, presumably due to the lower viscous friction with the microchannel walls and better conditions for acoustic wave propagation. Furthermore, the data indicates that the phase delay optimally designed between the sub-fingers of the CMUT array may shift to other values due to changes in the conditions affecting the propagation of acoustic waves, friction forces, radiation forces, wave reflections, and other effects not considered during the design, which might have a small, non-dominant effect on the device’s function. At optimum conditions, we were able to reach acoustic streaming velocities of over 2 mm/s, which is superior to any other reported numbers for microchannels with directly integrated CMUTs.

## Data Availability

The data presented in this study are available upon request from the corresponding authors.

## References

[1] Fan Y., Wang X., Ren J., Lin F., Wu J. (2022). Recent Advances in Acoustofluidic Separation Technology in Biology. Microsyst. Nanoeng..

[2] Afsaneh H., Mohammadi R. (2022). Microfluidic Platforms for the Manipulation of Cells and Particles. Talanta Open.

[3] Zhang P., Bachman H., Ozcelik A., Huang T.J. (2020). Acoustic Microfluidics. Annu. Rev. Anal. Chem. Palo Alto Calif..

[4] Pelenis D., Barauskas D., Sapeliauskas E., Vanagas G., Mikolajunas M., Virzonis D. (2017). Acoustical Streaming in Microfluidic CMUT Integrated Chip Controls the Biochemical Interaction Rate. J. Microelectromechanical Syst..

[5] Pelenis D., Barauskas D., Vanagas G., Dzikaras M., Viržonis D. (2019). CMUT-Based Biosensor with Convolutional Neural Network Signal Processing. Ultrasonics.

[6] Wu J. (2018). Acoustic Streaming and Its Applications. Fluids.

[7] Dong J., Liang D., Yang X., Sun C. (2021). Influences of Microparticle Radius and Microchannel Height on SSAW-Based Acoustophoretic Aggregation. Ultrasonics.

[8] Doinikov A.A., Thibault P., Marmottant P. (2018). Acoustic Streaming Induced by Two Orthogonal Ultrasound Standing Waves in a Microfluidic Channel. Ultrasonics.

[9] McLean J., Degertekin F.L. (2004). Directional Scholte Wave Generation and Detection Using Interdigital Capacitive Micromachined Ultrasonic Transducers. IEEE Trans. Ultrason. Ferroelectr. Freq. Control.

[10] Zhu J., Popovics J.S., Schubert F. (2004). Leaky Rayleigh and Scholte Waves at the Fluid–Solid Interface Subjected to Transient Point Loading. J. Acoust. Soc. Am..

[11] Sun L., Lehnert T., Gijs M., Li S. (2022). Polydimethylsiloxane Microstructure-Induced Acoustic Streaming for Enhanced Ultrasonic DNA Fragmentation on a Microfluidic Chip. Lab. Chip.

[12] Tong T., Yue S., Li R., Lin F., Chen D., Xing X., Chu W.-K., Song G., Liu D., Wang Z. (2022). Microfluidic Pumps with Laser Streaming from Tips of Optical Fibers and Sewing Needles. Adv. Opt. Mater..

[13] Samarasekera C., Sun J.G.W., Zheng Z., Yeow J.T.W. (2018). Trapping, Separating, and Palpating Microbead Clusters in Droplets and Flows Using Capacitive Micromachined Ultrasonic Transducers (CMUTs). Sens. Actuators B Chem..

[14] Vanagas G., Barauskas D., Virzonis D. Study of the CMUT Operation in Microfluidic Application. Proceedings of the 2012 IEEE International Ultrasonics Symposium.

[15] Joergensen J.H., Bruus H. (2021). Theory of Pressure Acoustics with Thermoviscous Boundary Layers and Streaming in Elastic Cavities. J. Acoust. Soc. Am..

[16] Devendran C., Collins D.J., Neild A. (2022). The Role of Channel Height and Actuation Method on Particle Manipulation in Surface Acoustic Wave (SAW)-Driven Microfluidic Devices. Microfluid. Nanofluidics.

[17] Lin L., Dang H., Zhu R., Liu Y., You H. (2022). Effects of Side Profile on Acoustic Streaming by Oscillating Microstructures in Channel. Micromachines.

